# Epigenetic prediction of complex traits and mortality in a cohort of individuals with oropharyngeal cancer

**DOI:** 10.1186/s13148-020-00850-4

**Published:** 2020-04-22

**Authors:** Ryan J. Langdon, Rhona A. Beynon, Kate Ingarfield, Riccardo E. Marioni, Daniel L. McCartney, Richard M. Martin, Andy R. Ness, Michael Pawlita, Tim Waterboer, Caroline Relton, Steven J. Thomas, Rebecca C. Richmond

**Affiliations:** 1 MRC Integrative Epidemiology Unit at the University of Bristol, Bristol, UK; 2 Population Health Sciences, Bristol Medical School, University of Bristol, Bristol, UK; 3 NIHR Bristol Biomedical Research Centre, University Hospitals Bristol and University of Bristol, Bristol, UK; 4 Centre for Trials Research, Neuadd Meirionnydd, Heath Park Way, Cardiff, UK; 5 Community Oral Health, University of Glasgow Dental School, Sauchiehall Street, Glasgow, UK; 6 MRC Human Genetics Unit, Institute of Genetics and Molecular Medicine, University of Edinburgh, Western General Hospital, Crewe Road, Edinburgh, Scotland, EH4 2XU UK; 7 Medical Genetics Section, Centre for Genomic and Experimental Medicine, Institute of Genetics and Molecular Medicine, University of Edinburgh, Edinburgh, EH4 2XU UK; 8 Infections and Cancer Epidemiology, German Cancer Research Center (DKFZ), Heidelberg, Germany

## Abstract

**Background:**

DNA methylation (DNAm) variation is an established predictor for several traits. In the context of oropharyngeal cancer (OPC), where 5-year survival is ~ 65%, DNA methylation may act as a prognostic biomarker. We examined the accuracy of DNA methylation biomarkers of 4 complex exposure traits (alcohol consumption, body mass index [BMI], educational attainment and smoking status) in predicting all-cause mortality in people with OPC.

**Results:**

DNAm predictors of alcohol consumption, BMI, educational attainment and smoking status were applied to 364 individuals with OPC in the Head and Neck 5000 cohort (HN5000; 19.6% of total OPC cases in the study), followed up for median 3.9 years; inter-quartile range (IQR) 3.3 to 5.2 years (time-to-event—death or censor). The proportion of phenotypic variance explained in each trait was as follows: 16.5% for alcohol consumption, 22.7% for BMI, 0.4% for educational attainment and 51.1% for smoking. We then assessed the relationship between each DNAm predictor and all-cause mortality using Cox proportional-hazard regression analysis. DNAm prediction of smoking was most consistently associated with mortality risk (hazard ratio [HR], 1.38 per standard deviation (SD) increase in smoking DNAm score; 95% confidence interval [CI] 1.04 to 1.83; *P* 0.025, in a model adjusted for demographic, lifestyle, health and biological variables). Finally, we examined the accuracy of each DNAm predictor of mortality. DNAm predictors explained similar levels of variance in mortality to self-reported phenotypes. Receiver operator characteristic (ROC) curves for the DNAm predictors showed a moderate discrimination of alcohol consumption (area under the curve [AUC] 0.63), BMI (AUC 0.61) and smoking (AUC 0.70) when predicting mortality. The DNAm predictor for education showed poor discrimination (AUC 0.57). *Z* tests comparing AUCs between self-reported phenotype ROC curves and DNAm score ROC curves did not show evidence for difference between the two (alcohol consumption *P* 0.41, BMI *P* 0.62, educational attainment *P* 0.49, smoking *P* 0.19).

**Conclusions:**

In the context of a clinical cohort of individuals with OPC, DNAm predictors for smoking, alcohol consumption, educational attainment and BMI exhibit similar predictive values for all-cause mortality compared to self-reported data. These findings may have translational utility in prognostic model development, particularly where phenotypic data are not available.

## Background

Peripheral blood DNA methylation (DNAm), which is a type of epigenetic modification, has been established as a predictor of complex health and lifestyle factors, and may be used to complement and refine self-reported phenotypes by circumventing issues of recall biases and potentially improving phenotypic resolution [1]. Multiple examples of the utility of DNAm for trait prediction exist in the epidemiological literature. Peripheral blood DNAm has been shown to serve as both a sensitive and specific biomarker of tobacco smoke exposure, with methylation status at one cytosine-phosphate-guanine (CpG) site in the aryl hydrocarbon receptor repressor (*AHRR*) gene (cg05575921) having a predictive area under the receiver operating characteristic curve (AUC) for smoking status of 0.99 for current vs never smokers [2]. Moreover, previous studies have found that peripheral blood DNAm at smoking-related CpG sites, both individually and in combination in ‘scores’ (methylation values derived from a weighted average of multiple trait-associated CpG sites), may have potential for improving lung cancer risk and mortality prediction over and above self-reported smoking information [3–6]. DNAm risk scores of other lifestyle characteristics, including alcohol consumption, body mass index (BMI) and educational attainment, have recently been developed in large training datasets and have been shown to independently explain a range of phenotypic variance, from 2.5% for educational attainment to over 60% for smoking [4]. These too have been shown to serve as predictors of disease outcomes, in addition to all-cause mortality in (healthy) population-based cohort studies [7, 8].

To date, the added prognostic utility of DNAm predictors in estimating mortality risk in clinical cohorts of individuals diagnosed with disease has not been thoroughly investigated. In the setting of a large prospective head and neck cancer cohort (the Head and Neck 5000 Study [9]), we attempted to use peripheral blood DNAm and self-reported data associated with four complex exposure traits of interest—alcohol consumption, smoking, BMI and educational attainment—to assess whether externally derived DNAm risk scores could provide an accurate prediction of phenotype in a subset of participants with oropharyngeal tumours. We also assessed the validity of these DNAm risk scores as biomarkers of mortality after a median 3.9 years follow-up (time-to-event [death or censor], inter-quartile range [IQR] 3.3 to 5.2 years), given that the four exposure traits of interest have been shown to be related to head and neck cancer (HNC) mortality in previous studies [10–16]. The peripheral blood DNAm risk scores were then compared with the self-reported measures of the four exposure traits in terms of their predictive ability.

## Results

The primary analysis included 364 individuals with DNAm and complete phenotypic and covariate data available. The baseline descriptive statistics of included participants are presented in Table 1 and stratified by human papillomavirus (HPV) status in Supplementary Table 1. In total, 78 of the 364 individuals died during a median follow-up period of 3.9 years (IQR 3.3 to 5.2). The Kaplan-Meier survival curves for mortality based on our covariates of interest are shown in Supplementary Figures 1a and b.

**Table 1:** Baseline descriptive statistics of included participants (*N* = 364)

	Alive (*N* = 273)		Dead (*N* = 91)		
Characteristic	*N*	Frequency	*N*	Frequency	*P* value
**Gender**					
Male	209	76.6%	75	82.4%	0.242
Female	64	23.4%	16	17.6%	
**Age at enrolment**					
< 44	20	7.3%	3	3.3%	0.016
45 to 54	83	30.4%	22	24.2%	
55 to 64	113	41.4%	34	37.4%	
65 to 74	48	17.6%	22	24.2%	
75 +	9	3.3%	10	11.0%	
**TNM stage**					
Low (I–II)	39	14.3%	8	8.8%	0.176
High (III–IV)	234	85.7%	83	91.2%	
**HPV status**					
Negative	61	22.3%	48	52.7%	< 0.001
Positive	212	77.7%	43	47.3%	
**BMI group**					
Not overweight	73	38.0%	31	55.4%	0.021
Overweight or obese	119	62.0%	25	44.6%	
**Comorbidity**^**a**^					
None	164	60.1%	34	37.4%	< 0.001
Mild	73	26.7%	29	31.9%	
Moderate/severe	36	13.2%	28	30.8%	
**Education level**					
School education	116	42.5%	45	49.5%	0.470
College	111	40.7%	34	37.4%	
Degree	46	16.8%	12	13.2%	
**Self-reported smoking status**					
Never	96	35.2%	11	12.1%	< 0.001
Former	140	51.3%	49	53.8%	
Current	37	13.6%	31	34.1%	
**Self-reported alcohol intake**					
Non-drinker	75	27.5%	22	24.2%	0.119
Moderate	68	24.9%	15	16.5%	
Hazardous-harmful	130	47.6%	54	59.3%	

*BMI* body mass index, *HPV* human papillomavirus, *N* number.^a^Comorbidity was defined using the Adult Comorbidity Evaluation-27 (ACE-27) index [37]. For the purposes of analysis, moderate and severe comorbidity groups were combined

### Proportion of phenotypic variance explained for DNAm-based risk scores

We generated five DNAm scores for alcohol consumption, two for BMI, one DNAm score for educational attainment and five for smoking, based on several large epigenome wide associations studies (EWAS), as outlined in Tables 2, 3, 4 and 5. The phenotypic variance explained by all DNAm risk scores is shown in Table 6. Where available, the Bayesian-derived DNAm risk scores for BMI and smoking [17] (BMI 24.5%, smoking 48.7%) explained a higher proportion of variance than least absolute shrinkage and selection operator-derived (LASSO-derived) (BMI 22.2%, smoking 43.5%) and generalised linear model-derived (glm-derived) (BMI N/A, smoking 40.5%) alternatives. The DNAm risk score for educational attainment (McCartney et al. [4]; LASSO model) explained the least variance of our phenotypes, at 0.43%. Finally, the DNAm risk score explaining the highest proportion of phenotypic variance in alcohol was derived from an EWAS meta-analysis using a LASSO model which gave the minimum cross-validated error (Liu et al. [18] model 4 16.5%).

**Table 2:** Origins of alcohol consumption DNAm scores employed in the current analysis

Phenotype	Origin publication	EWAS model	# CpG sites
Alcohol consumption	‘A DNA methylation biomarker of alcohol consumption’ Liu et al. [18]	EWAS (450 K) were conducted initially using linear models per cohort. Next, an inverse variance-weighted random-effects model was used to meta-analyse 8 European-ancestry cohorts. CpGs from the meta-analysis were taken forward and included in a least absolute shrinkage and selection operator (LASSO) regression in an independent cohort, with four selection criteria used to select CpGs with predictive value of alcohol consumption	Model 1: 5, model 2: 23, model 3: 78, model 4: 144
	‘Epigenetic prediction of complex traits and death’ McCartney et al. [4]	EWAS (MethylationEPIC) were conducted using a LASSO regression model with k-fold (*k* = 10) cross-validation.	450

**Table 3:** Origins of BMI DNAm scores employed in the current analysis

Phenotype	Origin publication	EWAS model	# CpG sites
BMI	‘Epigenetic prediction of complex traits and death’ McCartney et al. [4]	EWAS (MethylationEPIC) were conducted using a LASSO regression model with k-fold (*k* = 10) cross-validation.	1109
	‘Bayesian reassessment of the epigenetic architecture of complex traits’ Trejo Banos et al. [17]	EWAS (MethylationEPIC) were conducted using a Bayesian framework.	144

**Table 4:** Origins of educational attainment DNAm scores employed in the current analysis

Phenotype	Origin publication	EWAS model	# CpG sites
Educational attainment	‘Epigenetic prediction of complex traits and death’ McCartney et al. [4]	EWAS (MethylationEPIC) were conducted using a LASSO regression model with k-fold (*k* = 10) cross-validation.	373

**Table 5:** Origins of smoking DNAm scores employed in the current analysis

Phenotype	Origin publication	EWAS model	# CpG sites
Smoking	‘Epigenetic Signatures of Cigarette Smoking’ Joehanes et al. [19]	Linear mixed models were conducted, then combined in a random-effects model meta-analysis (450 K). After meta-analysis, one set of CpGs was selected based on a Bonferroni *P* value of *P* < 1 × 10^−7^ (485,381 tests) and another was selected based on a genome-wide false discovery rate *P* value < 0.05.	Bonferroni model: 2623, FDR model: 18760
	‘Self-reported smoking, serum cotinine, and blood DNA methylation’ Zhang et al. [20]	An EWAS (450 K) of cotinine concentration was conducted using median quantile regression, then CpG sites were individually validated against estimated average cigarettes per day using restricted cubic spline regression. Results were filtered by optimising AUCs derived from logistic regression for smoking status (current vs never; former vs never).	4
	‘Bayesian reassessment of the epigenetic architecture of complex traits’ Trejo Banos et al. [17]	EWAS (MethylationEPIC) were conducted using a Bayesian framework.	59
	‘Epigenetic prediction of complex traits and death’ McCartney et al. [4]	EWAS (MethylationEPIC) were conducted using a LASSO regression model with k-fold (*k* = 10) cross-validation.	233

**Table 6:** Proportions of phenotypic variance explained by the DNAm risk scores employed

Methylation score	Variance explained in phenotype
Smoking	
Trejo Bayesian (59 CpG sites)	48.7%
*AHRR* (cg05575921)	47.0%
McCartney LASSO (233 CpG sites)	43.5%
Joehanes (Bonferroni) (2623 CpG sites)	40.5%
Joehanes (FDR) (18,670 CpG sites)	33.5%
Zhang (4 CpG sites)	5.2%
Alcohol	
Liu model 4 (144 CpG sites)	16.5%
Liu model 3 (78 CpG sites)	15.8%
Liu model 1 (5 CpG sites)	13.9%
Liu model 2 (23 CpG sites)	10.3%
McCartney LASSO (450 CpG sites)	10.0%
BMI	
Trejo Bayesian (144 CpG sites)	24.5%
McCartney LASSO (1109 CpG sites)	22.2%
Educational attainment	
McCartney LASSO (373 CpG sites)	0.4%

### Relationship between self-reported phenotype and mortality

The multivariable Cox proportional-hazard outputs for minimally adjusted and fully adjusted models are presented in Table 7. In minimally adjusted models (adjusted for age and sex), smoking and alcohol intake were positively associated with mortality (HR 3.29, 95% CI 1.75 to 6.18, *P* 2.2. × 10^−4^ for ever versus (vs) never smokers and HR 1.62, 95% CI 1.06 to 2.49, *P* 0.027 for hazardous-to-harmful drinkers vs non-hazardous-to-harmful drinkers). BMI appeared to be protective (HR 0.93, 95% CI 0.87 to 0.99, *P* 0.028 × 10^−2^ for overweight vs not overweight). Educational attainment was not associated with mortality (HR 0.81, 95% CI 0.54 to 1.22, *P* 0.32 for higher education vs school education).

**Table 7:** Association of phenotypic and DNAm-based predictors of smoking, alcohol drinking, BMI and education with mortality

		Minimally adjusted^a^						Fully adjusted^b^			
Exposure	***N***	HR	ll	ul	***P*** value		***N***^c^	HR	ll	ul	***P*** value
Self-reported phenotype											
Ever vs never smoker	364	3.29	1.75	6.18	2.22 × 10^−4^		364	2.21	1.14	4.30	0.019
Hazardous to harmful drinker vs not	364	1.62	1.06	2.49	0.027		364	1.34	0.86	2.09	0.202
Higher education vs school education	364	0.81	0.54	1.22	0.320		364	0.87	0.57	1.31	0.503
BMI	248	0.93	0.87	0.99	0.028		248	0.98	0.92	1.06	0.664
DNAm score											
*Smoking*											
McCartney LASSO (233 CpG sites)	364	1.53	1.24	1.88	7.89 × 10^-5^		364	1.20	0.94	1.52	0.144
Trejo Bayesian (59 CpG sites)	364	1.70	1.37	2.11	1.49 × 10^-6^		364	1.26	0.93	1.72	0.140
*AHRR* (cg05575921)	364	0.59	0.48	0.74	1.72 × 10^-6^		364	0.79	0.58	1.07	0.125
Joehanes (FDR) (18,760 CpG sites)	364	1.70	1.34	2.15	1.27 × 10^−5^		364	1.35	0.99	1.84	0.056
Joehanes (Bonferroni) (2623 CpG sites)	364	1.67	1.36	2.05	7.57 × 10^−7^		364	1.38	1.04	1.83	0.025
Zhang (4 CpG sites)	364	1.48	1.16	1.88	1.48 × 10^−3^		364	1.28	1.02	1.60	0.036
*Alcohol*											
Liu (5 CpG sites)	364	1.32	1.10	1.57	2.50 × 10^−3^		364	1.19	0.97	1.47	0.094
Liu (23 CpG sites)	364	1.26	1.04	1.52	0.019		364	1.10	0.89	1.36	0.357
Liu (78 CpG sites)	364	1.25	1.07	1.45	5.02 × 10^−3^		364	1.20	0.99	1.45	0.067
Liu (144 CpG sites)	364	1.24	1.07	1.44	5.31 × 10^−3^		364	1.21	1.00	1.46	0.052
McCartney LASSO (450 CpG sites)	364	1.28	1.03	1.60	0.024		364	1.05	0.79	1.41	0.723
*BMI*											
Trejo Bayesian (144 CpG sites)	364	0.78	0.63	0.97	0.024		248	0.77	0.56	1.08	0.132
McCartney LASSO (1109 CpG Sites)	364	0.85	0.68	1.06	0.146		248	0.77	0.57	1.04	0.093
*Education*											
McCartney LASSO (373 CpG sites)	364	0.76	0.61	0.96	0.021		364	0.87	0.68	1.12	0.270

*N* number, *HR* hazard ratio, *ll* lower confidence interval, *ul* upper confidence interval. ^a^Self-reported phenotypes adjusted for age and gender; epigenetic scores adjusted for age, gender, cell counts and batch effects. ^b^Phenotypes additionally adjusted for clinical variables (TNM stage, HPV status and comorbidity), and a combination of smoking, alcohol intake, education and BMI, as appropriate to the model; risk scores additionally adjusted for clinical variables, the corresponding phenotype predicted by the score of interest and the remaining self-reported phenotypes (excluding BMI). ^c^Sample numbers vary due to missing BMI data

The association of self-reported smoking status with mortality remained (albeit attenuated) following adjustment for demographic (age, sex), clinical (TNM stage, HPV status, comorbidity) and phenotypic (alcohol consumption and education) variables (HR 2.21, 95% CI 1.14 to 4.30, *P* 0.019). Analogous results were observed in the imputed analysis (to account for covariate missingness; Supplementary Table 2), with smoking being the only phenotype associated with mortality in the fully adjusted models (HR 2.56, 95% CI 1.30 to 4.92, *P* 4.9 × 10^−3^).

### Relationship between DNAm scores and mortality

All the DNAm risk scores were related to mortality in the minimally adjusted models (adjusted for age, sex, cell counts and batch effects) (Table 7), except for the BMI predictor derived by McCartney et al. [4]. After adjusting for clinical factors and self-reported phenotypes, the smoking-derived DNAm scores developed by Joehanes et al. (Bonferroni) [19] and Zhang et al. [20] were most strongly associated with mortality risk (Joehanes et al. HR 1.38, 95% CI 1.04 to 1.83, *P* 0.025; Zhang et al. HR 1.28, 95% CI 1.02 to 1.60, *P* 0.036), with some evidence of association also found for the Liu et al. alcohol-derived DNAm score (144 CpG sites) (HR 1.21, 95% CI 1.00, 1.46, *P* 0.052). There was a modest positive correlation between the phenotypic variance explained by the various DNAm scores and their magnitude of association with mortality (Supplementary Figure 2; *R*^2^ = 0.29), with some outliers. For example, the Zhang et al. predictor of smoking explained 5.2% phenotypic variance (the lowest out of the DNAm predictors of smoking) but showed the third-highest absolute HR for OPC mortality of the 6 smoking DNAm predictors (HR 1.28, Table 7—fully adjusted).

### Predictive accuracy of DNAm risk scores against mortality

Given the largest amount of phenotypic variance explained, Bayesian DNAm risk scores for BMI and smoking were used to predict mortality. For the same reason, the DNAm risk derived from McCartney et al. [4] was used as a predictor for educational attainment and the DNAm risk from Liu et al. [18] (model 4) was used as a predictor for alcohol consumption.

Across all four phenotypes assessed in our study, the AUC when DNAm risk scores were used to predict mortality was greater than self-reported phenotypes (Supplementary Figure 3), although the difference was modest (*Z* test *P* value for comparison of DNAm AUC and self-reported AUC for the following: smoking = 0.19, alcohol = 0.41, BMI = 0.62, educational attainment = 0.49). When a generalised linear model of DNAm risk score and corresponding self-reported phenotype were used to predict mortality, the AUC improved over self-reported phenotype alone, but again with only modest improvement (*Z* test *P* value for combined epigenetic risk score and self-reported phenotype AUC vs self-reported phenotype AUC for the following: smoking = 0.30, alcohol = 0.38, BMI = 0.71, educational attainment = 0.26). The most predictive epigenetic risk score for mortality was that of smoking, with an AUC of 0.70 (vs 0.67 for self-report). The weakest epigenetic risk score predictor against mortality was our predictor of educational attainment, with an AUC of 0.57 (vs 0.54 for self-report).

### Sensitivity analysis

A summary of the baseline descriptive characteristics of participants included in the sensitivity analysis is provided in Supplementary Table 3. When the analysis was restricted to participants with data available for BMI (*N* = 248) (Supplementary Table 4), the results of models examining the association of self-reported phenotypes with mortality followed a similar trend; only self-reported smoking was associated following full adjustment (adjusted for age, sex, TNM stage, HPV status, comorbidity and a combination of smoking, alcohol intake, education and BMI, as appropriate to the model).

When the relationships between DNAm scores and mortality were examined, there was evidence that all alcohol consumption DNAm scores derived from Liu et al. were associated with mortality (5 CpG score [most associated] HR 1.36, 95% CI 1.08 to 1.73, *P* 9.39 × 10^−3^), in addition to the Bayesian score for BMI (HR 0.76, 95% CI 0.59 to 0.99, *P* 0.045). For the smoking DNAm scores, the Joehanes et al. (HR 1.84, 95% CI 1.36 to 2.49, *P* 7.43 × 10^−5^), McCartney et al. (HR 1.49, 95% CI 1.13 to 1.97, *P* 4.31 × 10^−3^), Zhang et al. (HR 1.41, 95% CI 1.04 to 1.91, *P* 0.029), *AHRR* (HR 0.63, 95% CI 0.47 to 0.83, *P* 1.28 × 10^−3^) and Bayesian scores (HR 1.61, 95% CI 1.21 to 2.14, *P* 1.17 × 10^−3^) showed evidence of association with mortality (Supplementary Table 4).

Following full adjustment (as for self-reported phenotypes, additionally adjusted for cell counts and batch effects), three Liu et al. alcohol DNAm scores remained associated with mortality (5 CpG score HR 1.43, 95% CI 1.07 to 1.92, *P* 0.017, 78 CpG score HR 1.32, 95% CI 1.03 to 1.69, *P* 0.027, 144 CpG score HR 1.29, 95% CI 1.02 to 1.63, *P* 0.036). Additionally, three smoking DNAm scores remained associated with mortality (Joehanes [FDR] (18,760 CpGs) HR 1.59, 95% CI 1.09 to 2.32, *P* 0.016, Joehanes [Bonferroni] (2623 CpGs) HR 1.50, 95% CI 1.06 to 2.12, *P* 0.022, Zhang HR 1.33, 95% CI 1.00 to 1.77, *P* 0.047) (Supplementary Table 4).

Analogous results for our minimally and fully adjusted Cox regression HRs between DNA and mortality were obtained in an imputed analysis (*N* = 408) (Supplementary Table 5). There was additional evidence of a relationship between *AHRR* methylation status and mortality in the imputed analysis, whereby a SD unit decrease in cg05575921 methylation (smoking is associated with hypomethylation at this loci) was associated with a 26% decrease in risk of death (HR 0.74, 95% CI 0.56 to 0.98, *P* 0.033) in the fully adjusted model (model 4). There was also an association between all-cause mortality and the Bayesian-derived DNAm risk score for BMI in the imputed analysis (fully adjusted HR 0.72, 95% CI 0.56 to 0.91, *P* 7.24 x 10^-3^).

## Discussion

We estimated the predictive accuracy of thirteen DNAm risk scores for smoking, alcohol consumption , BMI and educational attainment, in comparison with self-reported phenotypes. We then used these DNAm scores to assess mortality risk in a clinical cohort of individuals with oropharyngeal cancer , using a Cox proportional-hazard model.

The maximum proportion of phenotypic variance explained in each trait by any one DNAm score was as follows: 16.5% for alcohol consumption, 24.5% for BMI, 0.4% for educational attainment and 48.7% for smoking. All phenotypes proxied by a DNAm risk score yielded similar mortality estimates to those of self-reported phenotypes. Results from our fully adjusted model show that self-reported smoking is the only trait strongly associated with mortality risk after adjustment for age, sex, TNM stage, HPV status, comorbidity, alcohol consumption and educational attainment. Similarly, DNAm prediction of smoking was most consistently associated with mortality risk after adjusting for clinical factors and self-reported phenotypes, with some evidence of association for the alcohol and BMI DNAm scores. DNAm predictors explained similar levels of variance in mortality to self-reported phenotypes. ROC curves for the DNAm predictors showed a moderate discrimination of alcohol consumption, BMI and smoking when predicting mortality. The DNAm predictor for education showed poor discrimination. Results provided evidence for a gain of 0.03 in AUC but power was limited to detect a statistical improvement in prediction given the small number of deaths. *Z* tests comparing AUCs between self-reported phenotype ROC curves and DNAm score ROC curves did not show evidence for difference between the two.

Smoking has been shown to be an independent prognostic factor for OPC in prospective studies [21], case-control studies [22] and systematic reviews [23]. Beynon et al. investigated the wider HN5000 cohort (*N* = 1393, oral cavity cancer *N* = 403, oropharyngeal cancer *N* = 660, laryngeal cancer *N* = 330) for the prognostic value of self-reported smoking and alcohol consumption, finding that only smoking influenced all-cause mortality in models adjusted for age, gender, ethnicity, stage, comorbidity, BMI, HPV status, treatment, education, deprivation index, income, marital status and either smoking or alcohol use [24]. Moreover, Beesley et al. investigated the prognostic value of existing OPC ‘calculators’ developed between 2003 and 2016 [25]. Four such calculators were evaluated, derived from Maastro Clinic data [26], Radiation Therapy Oncology Group (RTOG) trial data [27], patient data from eastern Denmark [28] and Erasmus Medical Centre data [29]. Three of these calculators (Maastro Clinic, RTOG and Denmark) include pack-years of smoking as a prognostic variable; none of them include a metric of alcohol consumption.

For the prediction of mortality using DNAm scores, the two predictors that were derived using a Bayesian framework (smoking and BMI) explained the most phenotypic variance and were therefore employed over other epigenetic scores derived using a LASSO/linear mixed-effects regression. Despite explaining the largest amount of phenotypic variance, neither Bayesian predictor was associated with mortality as strongly as their respective directly measured phenotype. One potential explanation for this finding is that the LASSO/linear mixed-effects-derived DNAm scores capture elements of smoking and BMI, respectively, which are more associated with mortality (e.g. smoking heaviness or visceral fat mass), whereas the Bayesian-derived DNAm scores may be a more composite measure of phenotype and better predict it.

In our minimally adjusted models, self-reported BMI and alcohol consumption, DNAm risk scores for alcohol consumption and a DNAm risk score for education all showed evidence of an association with mortality. However, when we adjusted for clinical covariates and mutually adjusted for the four exposure phenotypes in our fully adjusted models, the associations notably attenuated. This could reflect over -adjustment i.e. by adjusting for mediators which actually lie on the causal pathway between phenotype and mortality. Additionally, adjusting for variables which are strongly correlated (i.e. by including both self-report and DNAm scores for the same phenotype in the same model) can lead to imprecision in the effects estimated by our regression models. However, in the case of prediction, it is precisely the added value of the DNAm score over and above the phenotype which we were interested in estimating, hence the choice of variables in our fully adjusted models.

Most of the attenuation in the strength of association between self-reported phenotypes and mortality came with adjustment for clinical variables (model 2 in Supplementary Table 2), whilst for the methylation scores, adjusting for the corresponding self-reported phenotypes led to the biggest attenuation in estimates (model 3 in Supplementary Table 2). Nonetheless, there was evidence of an association between the smoking and, to a lesser extent, the alcohol consumption and BMI DNA methylation scores with mortality, even in the fully adjusted model, which may reflect the true effects of the corresponding phenotypes on OPC mortality in our study.

This study has several strengths including the availability of Illumina MethylationEPIC array data and the availability of DNAm risk scores derived from large-scale studies (see Supplementary Table 6). As the MethylationEPIC platform supersedes the older Illumina 450 K array and provides ~ 400,000 more CpG sites to interrogate in relation to a phenotype (whilst maintaining the vast majority of sites already on the 450 K array), the DNAm risk scores derived from this platform (and applied to our data) explain a greater proportion of phenotypic variance than those derived from the 450 K array (Tables 2, 3, 4, 5 and, 6).

The availability of DNAm data and comprehensive mortality follow-up data in the same cohort, as well as our ability to adjust for multiple biological, clinical and lifestyle covariates, including HPV status, presents another major strength of our study. It enabled investigation of the association of DNAm scores with mortality within a cancer cohort—a novel application of epigenetic prediction which may have clinical utility in the future.

A notable limitation of our analysis is the small sample size with a relatively limited number of deaths. Additionally, our models examining the effect of BMI on mortality risk are not directly comparable to those estimating the mortality risk associated with smoking, drinking and education, as the included populations would differ due to missing data. We used multiple imputation (MI) techniques in our sensitivity analysis to address this issue, as ignoring missing data, or failing to adequately account for it can lead to bias and a loss of precision in parameter estimates [30]. The most common approach for addressing missing data (and the default in most statistical packages) is complete case analysis [31]. However, a major disadvantage of complete case analysis, particularly in smaller sample sizes, is that it can diminish statistical power through simply discarding samples with incomplete data. If BMI had been included as a covariate in our fully adjusted models, this would have reduced the statistical power, shown by the loss of precision in our complete case sensitivity analyses. Conversely, MI makes use of all the available data, but under the assumption that data is missing completely at random (MCAR) or missing at random (MAR). When data are missing not at random, complete case analysis gives the most unbiased results [31]. In the case of BMI, which had the most missing data, the baseline descriptive characteristics of participants with or without data on this variable did not appear to be different, presumably because BMI data was MCAR. Accordingly, the MI approach adopted is likely to be valid and provides further support for our findings.

Another limitation of our study is that we were only able to assess all-cause mortality, as cause-of death data were not available for all participants in the current HN5000 data release. Moreover, previous work has shown that the cause of death information on a death certificate is often inaccurate [32, 33]. Whilst all-cause mortality will be impacted by cancer status, it will not show specificity to OPC as deaths could arise from competing causes such as cardiovascular disease, secondary cancers or age, preventing us from estimating phenotype risk on OPC-specific death. However, hazard ratio estimates are larger in our analysis compared to another study examining the association of DNAm scores against mortality in a healthy population. McCartney et al. [4] report a HR per SD increase in score of 1.29 (95% CI 1.05 to 1.57, *P* 0.013) for their smoking DNAm risk score (vs our HR per SD increase in smoking DNAm score 1.72, 95% CI 1.21 to 2.45, *P* 2.50 × 10^−3^, two-sided *Z* test *P* 0.21). All-cause mortality estimates in those with OPC likely reflect the effect of sustained heavy tobacco and alcohol use (a hallmark demographic of HNC populations), in addition to presence of cancer. The difference in mortality estimates may therefore reflect the effect these behaviours have on DNAm patterns, potentially correlating with an increase in proportion of phenotypic variance explained by DNAm in these prognostic factors and allowing clearer distinction between those dead vs alive, compared to a healthy population. In published literature, notable changes in DNAm have been reported in response to smoking [34], alcohol consumption [35], OPC oncogenesis and progression [36]. The marked HR differences seen between those with and without OPC illustrate a need to separately risk-stratify those with the disease from those without.

## Conclusion

In summary, we have shown that in the context of OPC, peripheral blood DNAm-based scores are able to predict complex traits with a relatively high proportion of variance explained for smoking, alcohol consumption and BMI; but not educational attainment. Comparing the effect on mortality of both peripheral blood DNAm predictors and self-reported phenotype yielded similar results, with peripheral blood DNAm displaying similar effects on mortality across all traits assessed. Our findings suggest peripheral blood DNAm predictors can be used to supplement a prediction model of mortality in those with oropharyngeal cancer, potentially providing reliable insight into smoking, alcohol consumption and BMI measures in situations where self-reported phenotype information is not available for these individuals.

## Methods

### Study population

The study population for this analysis was drawn from individuals enrolled in the Head and Neck 5000 clinical cohort study (HN5000) [9]. Full details of the study methods and overall population are described in detail elsewhere [9, 37]. Briefly, between April 2011 and December 2014, 5511 individuals with HNC were recruited from 76 centres across the UK. All people with a new diagnosis of HNC were eligible to join the study and were recruited before or within a month of their cancer treatment commencing. Individuals with cancers of the pharynx, mouth, larynx, salivary glands and thyroid were included, whilst those with lymphoma, tumours of the skin or a recurrence of a previous head and neck cancer were excluded from the study. The study is estimated to have captured a third of all incident cases in the UK at the time of enrollment.

Local research nurses obtained informed consent from individuals, which included agreement to: collect, store and use biological samples; obtain samples of stored tissue; carry out genetic analyses and collect information from hospital notes and through record linkage. Ethics approval for this study was granted by the National Research Ethics Committee (South West Frenchay Ethics Committee, reference [10] /H0107/57, November 5, 2010) and approved by the research and development departments from participating National Health Service (NHS) Trusts.

**Fig. 1:**
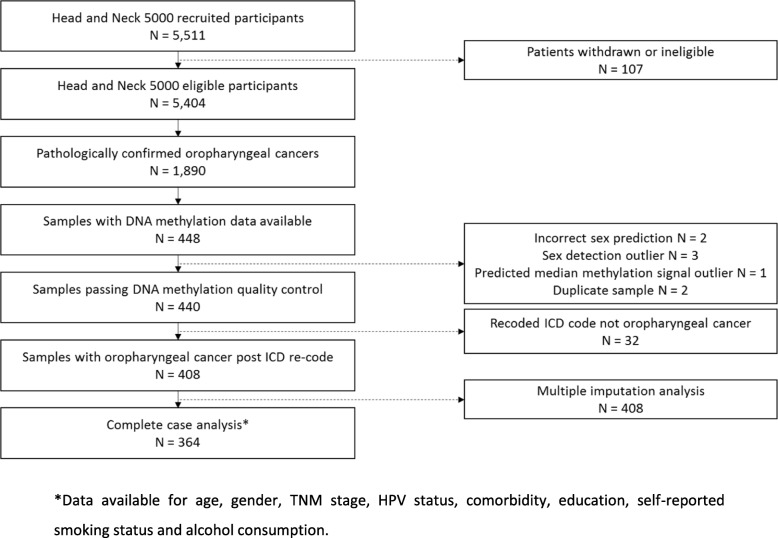
Flow diagram of HN5000 participants included in the analysis. *Data available for age, gender, TNM stage, HPV status, comorbidity, education, self-reported smoking status and alcohol consumption

Participants for the current study were selected from the HN5000 cohort based on a hierarchy of the following: (i) an ICD-10 coding (pathological where available) of oropharynx (CO1, CO5, CO9, C10.0–2, C10.3, C10.8 and C10.9); (ii) availability of OncoChip genotype data generated previously [38]; (iii) baseline questionnaire and clinical information (diagnosis, treatment and comorbidity) and (iv) both blood and saliva samples taken at baseline (*N* = 448, 23.5% of all OPC in HN5000) (see Fig. 1).

### Baseline data collection

Participants were asked to complete a series of three self-administered questionnaires at baseline enquiring about the following: (1) social and economic circumstances, overall health and lifestyle behaviours; (2) physical and psychological health, well-being and quality of life and (3) past sexual history and behaviours [9]. Clinical information on diagnosis, treatment and comorbidity was recorded on a short data capture form using questions based on a national audit [39]. Diagnoses were coded using the International Classification of Diseases (ICD) version 10 [40] and clinical staging of the tumour was based on the American Head and Neck Society TNM staging [41]. Comorbidity was defined using the Adult Comorbidity Evaluation-27 (ACE-27) index [42]. Nurses graded participants’ comorbidities into one of four categories according to the severity or organ decompensation: none, mild, moderate, or severe. An overall comorbidity score was assigned according to the severity of the highest-ranked medical condition, except in cases with two or more grade 2 ailments in different organ systems, where a final score of three was assigned.

Research nurses collected a blood sample from all consenting participants [9]. These were then sent to the study centre laboratory https://www.bristol.ac.uk/population-health-sciences/research/groups/bblabs/ at ambient temperature for processing. The samples were shipped to the laboratory by the next available first-class post using the transfer kits provided. Over 60% of samples arrived within 48 h and over 85% within 72 h. The blood samples were centrifuged at 3500 rpm for 10 min and the buffy coat layer used for DNA extraction. Any additional samples from the same participant were frozen and stored at − 80 °C. DNA extraction was carried out by LGC genomics (http://www.lgcgenomics.com/) using the Kleargene spin column extraction method (http://www.lgcgroup.com/products/dna-extraction-kits/kleargene-spin). Samples were eluted in a 1-ml low salt buffer and DNA quantified using picogreen. The mean DNA concentration across all HN5000 samples was 97.21 ng/μl, (SD 46 ng/μl).

#### Assessment of tobacco, alcohol, BMI and education

Information on tobacco and alcohol consumption, highest educational obtainment and BMI was obtained from baseline questionnaires, which are available on the study website (http://www.headandneck5000.org.uk/). Smoking was defined as having smoked at least one daily cigarette during a whole year and current smoking status was defined as ‘current’, ‘former’ or ‘never’. Among smokers, information on smoking status, age at smoking initiation and number of years of smoking was obtained.

Respondents were asked to report their average weekly alcohol consumption of a range of beverage types (wine, spirits and beer/larger/cider) before their diagnosis of head and neck cancer. From these measures, we derived an average intake of alcohol consumption in units per week, where one alcohol unit was equal to 10 ml or 8 g of pure alcohol. Baseline drinking categories were then defined as none, moderate (men and women drinking < 14 units/week), hazardous (men consuming 14–50 units/week; women consuming 14–35 units/week) and harmful (men consuming > 50 units/week; women consuming > 35 units/week) [43].

BMI was calculated as weight (kg)/[height (m)]^2^ and was based on participants’ self-report. At the start of data collection, baseline questionnaires did not enquire about participants’ height and weight and as a result, BMI data are missing for just over 40% of participants overall. For those with available data, a BMI of ≤ 25 was classed as ‘not overweight’, a BMI of > 25–≤ 30 was classed as ‘overweight’ and a BMI > 30 was classed as ‘obese’. Participants’ highest educational attainment was defined as ‘school educated’, ‘college educated’ or ‘degree level’.

#### Study follow-up and mortality

Notification of cancer registrations and mortality among HN5000 cohort members were received from the NHS Central Register and NHS Digital (formerly known as the Health and Social care information Centre), through linkage via NHS numbers. The last person was recruited into HN5000 on December 31, 2014 and follow-up information on mortality status was obtained up to September 1, 2018. Median follow-up from cohort entry to death or censoring (end of follow-up for this analysis—September 1, 2018) was 3.9 years (IQR 3.3 to 5.2).

### Epigenetic profiling and pre-processing

 DNAm data from peripheral blood samples were generated on participants using Infinium MethylationEPIC BeadChips (Illumina, USA). Following extraction, DNA was bisulphite-converted using the Zymo EZ DNA MethylationTM kit (Zymo, Irvine, CA, USA). Epigenome-wide methylation data were generated using the MethylationEPIC array according to the manufacturer protocol. The arrays were scanned using an Illumina iScan (version 2.3). Raw data files (IDAT files) were pre-processed using the R package *meffil* (https://github.com/perishky/meffil/) [44] to perform quality control (QC) and normalisation, as described previously [45]. From the initial 448 samples available, 8 samples did not pass QC: 2 samples with incorrect sex prediction, 3 samples with sex detection outliers, 1 sample with an outlier in predicted median methylated vs unmethylated signal and 2 duplicate samples. An additional 32 individuals were subsequently removed from the analysis owing to pathological re-classification, leaving 408 participants with DNAm data available (Fig. 1). During QC, probe intensities were dye-bias and background corrected using the ‘noob’ method developed by Triche et al. [46]. A total of 3674 probes were excluded, leaving 863,289 CpGs with which to perform analyses—2704 probes were removed due to a high proportion of high detection *P* values (> 10% of samples with a detection *P* value > 0.1) and 970 CpGs had low bead numbers in a high proportion of samples (< 3 beads in > 10% samples). Following QC, we performed functional normalisation (originally developed by Fortin et al. [47]) using the *Meffil* R package, which exploits control probes to separate biological variation from technical variation. Data were normalised using 6 control probe principal components derived from technical probes. During the normalisation process, probe intensity quantiles were normalised between samples by fitting linear models to these 6 derived principal components. The resulting quantile residuals for each QC object were retained as a set of normalised quantiles and used in a second normalisation step, where the raw probe intensities for each sample were adjusted to conform to its own set of normalised quantiles. After the second step had been completed for each sample, the resulting normalised DNAm data subsets were merged into a single dataset for analysis.

Post-normalisation, estimation of blood cell proportions, per sample, were estimated via the Houseman cellular composition prediction algorithm [48]. We used a cell-type reference (Reinius et al. 2012 [49]) to estimate proportions of neutrophils, natural killer cells, B cells, eosinophils, CD4T cells, CD8T cells and monocytes.

### DNAm risk score generation

Peripheral blood DNAm scores for alcohol consumption, smoking, BMI and educational attainment were based on independently identified CpG sites from several large epigenome-wide association studies (*N* = 500 to 9643; see Supplementary Table 6 [4, 17–20];). Details of regression model, sample size, year of publication and number of CpGs for each EWAS used to derive DNAm risk scores are shown in Tables 2, 3, 4 and 5. For each individual, DNAm scores were calculated as the product-sum of the effect size for each CpG from the respective EWAS results, multiplied by the normalised methylation (beta) value (post-QC) of the same CpG site in the HN500 MethylationEPIC data. Beta values are the ratio of methylated probe intensity compared to the overall intensity (sum of methylated and unmethylated probe intensities).

### Statistical analysis

#### Associations of DNAm scores with self-reported phenotypes

We performed linear regression analyses, adjusted for age, sex, stage, cell counts and batch effects, of DNAm risk scores against self-reported data to determine which scores explained the largest amount of variance in our exposure phenotypes of interest. We used the *R*^2^ statistic generated by the ‘lm’ function of the core Stats package in R (v3.4.1) as our measure of variance explained. 

#### Survival analysis

The end point of this study was all-cause mortality, defined as the time in days from study enrolment to date of death from any cause, or the date of censorship (i.e. the last date of follow-up for this analysis 01/09/2018). The primary analyses included complete cases only, i.e. participants with complete data for all the covariates used in the adjusted models and DNAm data available. Kaplan-Meier curves and the log-rank test were first used to investigate the univariate impact of covariates on mortality. The proportional hazard assumption was checked using statistical tests and graphical diagnostics based on the Schoenfeld residuals. Mortality risk was assessed in relation to each of the self-reported phenotypes (i.e. for smoking, alcohol drinking, BMI and education level) and DNAm scores, using Cox proportional-hazard models. All DNAm scores from Tables 2, 3, 4 and 5 were standardised (z-scored) to allow direct comparison of effect sizes with each other. Hazard ratios (HRs) and 95% confidence intervals (CIs) for mortality were calculated for each standard deviation (SD) increase in these scores. The HRs represent the increase in mortality risk for ever versus never smokers, hazardous to harmful drinkers versus non-hazardous to harmful drinkers, higher education (college or degree-level) versus school education and the difference in mortality risk per unit increase in BMI.

To assess potential associations of the four self-reported exposure phenotypes with mortality we fitted three regression models: (1) a minimally adjusted model that controlled for age and sex; (2) a model that additionally adjusted for clinical factors (TNM stage, HPV status and comorbidity) and (3) a fully adjusted model that mutually adjusted for the other self-reported phenotypes of interest. The clinical factors were selected on the basis of the strength of prior evidence linking them with HNC survival. Higher TNM stage is consistently associated with poorer survival [50]. HPV positivity, despite being a risk factor for OPC (that is, tumours driven by HPV infection, in particular HPV16) confers a marked survival advantage to those with OPC without HPV-driven tumours [22]. Comorbidity greatly affects all-cause mortality in both general populations and cancer populations [51, 52]. Owing to missing data, models examining the associations of self-reported smoking, alcohol drinking and education with mortality were not adjusted for self-reported BMI (model 3) because this would have reduced the sample size by 148 individuals (and therefore, statistical power). Ethnicity was not included as a potential covariate in this study because the cohort is almost exclusively white (97.1%).

Four separate models were fit to examine the relationship between DNAm scores with mortality: (1) a minimally adjusted model that adjusted for age, sex, cell counts and batch effects; (2) a ‘clinical model’, as above; (3) a model that additionally adjusted for the corresponding self-reported phenotype (e.g. models that examined the association of smoking-related DNAm scores with mortality adjusted for self-reported smoking status) and (4) a model that additionally adjusted for the other self-reported phenotypes (excluding BMI to preserve sample numbers). Batch effects included the DNAm bisulphite conversion date and the MethylationEPIC array slide and position of each sample. Results of the minimally adjusted (model 1) and fully adjusted (model 4) models are presented. The outputs from models 2 and 3 can be found in the supplementary material.

It was decided a priori not to restrict the complete case analysis to participants with self-reported BMI data available due to the amount of missing data, as this would decrease the statistical power to detect an effect of our exposures on mortality. Therefore, as a sensitivity analysis, another dataset was analysed as above but with complete data for BMI (self-reported). Finally, the entire dataset was analysed using MI as described below.

#### Multiple imputation

Data were missing for age at consent (1.2%), BMI (33.3%), comorbidity (0.74%), highest education level obtained (4.7%), annual household income (13%), self-reported smoking status (3.9%) and self-reported alcohol consumption (1.97%) (Supplementary Table 7). Missing values were imputed using the ‘ICE’ package for multiple chained equations in STATA (version 15) [53]. MI assumes that data is either MCAR or MAR, in which case data are acknowledged to be missing for non-random reasons but the missingness can be accounted for by observed variables (e.g. people with high education tend not to disclose their income) [54]. Information on BMI was likely MCAR since this information was not collected at the start of the study and missing BMI data appeared to be unrelated to observed values of other variables. Twenty imputed datasets were generated and then combined using Rubin’s rule to obtain valid statistical inferences [55]. The imputation model included the event indicator, the Nelson-Aalen estimator of the cumulative hazard, all of the variables that were used in substantive Cox models and any other available variables that help to explain the missing data. Analysis of the stacked datasets was performed with the prefix command ‘mim’, to obtain combined parameter estimates [56].

#### Predictive accuracy of DNAm risk scores against mortality

To assess the accuracy with which the DNAm risk scores for phenotypes could independently predict mortality (rather than directly affect it as described in “Survival analysis” section), we derived ROC curves of DNAm risk scores as predictor variables and used all-cause mortality as a response variable, using the pROC R package [57]. AUC was computed using the trapezoidal rule. We calculated 3 ROC curves per phenotype (alcohol consumption, BMI, educational attainment, smoking):

1. The DNAm risk score which explained the largest variance in each phenotype
2. Self-reported phenotype as the predictor
3. A generalised linear model combining both epigenetic risk score of the phenotype and the self-reported phenotype

To assess whether prediction of mortality using an epigenetic risk score improved upon prediction of mortality using self-reported phenotype, we conducted *Z* tests to compare AUCs using the DeLong *Z* test [58, 59], using the pROC R package. We compared epigenetic risk score for phenotype against self-reported phenotype (1. vs 2. above), in addition to comparing the generalised linear model of both epigenetic risk score and self-reported phenotype against self-reported phenotype alone (3. vs 2. above).

## Supplementary information

**Additional file 1: Supplementary Table 1**: Baseline descriptive characteristics of included participants, stratified by HPV status. **Supplementary Table 2**: Multivariable Cox proportional hazards results for model 2 (clinical) and model 3 (respective phenotype). **Supplementary Table 3**: Baseline descriptive characteristics of participants included in the sensitivity analysis (n=248). **Supplementary Table 4:** Results of the sensitivity analysis restricted to participants with data available for BMI. **Supplementary Table 5**: A comparison of minimally adjusted and fully adjusted Cox proportional hazards models results, using the imputed dataset (n=408). **Supplementary Table 6**: Details of array type and sample size for studies used to derive DNAm scores in this analysis. Supplementary Table 7: Proportion of missing data (n=408).

**Additional file 2: Supplementary Figure 1a**: Kaplan-Meier survival curves based on demographic and clinical covariates. Comorbidity categories were defined according to the severity or organ decompensation: none (coded 0), mild (coded 1), moderate (coded 2), or severe (coded 3). See text for more details. **Supplementary Figure 1b**: Kaplan-Meier survival curves based on our phenotypes of interest. **Supplementary Figure 2**: The association between variance explained by DNAm score and hazard ratio for 4-year mortality (Model 3). Hazard ratios are plotted as absolute log-transformed values for comparability. **Supplementary Figure 3**: ROC curves detailing the predictive accuracy of DNAm risk scores, self-reported phenotype and a combination of the two, against ~4-year mortality (median 3.9 years) in HN5000. ROC curves are provided for smoking, alcohol consumption, BMI and educational attainment. DNAm AUCs reflect use of the DNAm scores for these phenotypes which explained the greatest phenotypic variance: smoking = Trejo Bayesian model, alcohol consumption = Liu et al. model 4, BMI = Trejo Bayesian model, educational attainment = McCartney LASSO model. **Abbreviations:** AUC, area under curve; DNAm, DNA methylation; ROC, receiver-operator curve

## Data Availability

This publication presents data from the Head and Neck 5000 study. The study was a component of independent research funded by the National Institute for Health Research (NIHR) under its Programme Grants for Applied Research scheme (RP-PG-0707-10034). The views expressed in this publication are those of the author(s) and not necessarily those of the NHS, the NIHR or the Department of Health. Human papillomavirus (HPV) serology was supported by a Cancer Research UK Programme Grant, the Integrative Cancer Epidemiology Programme (grant number: C18281/A19169).

## Abbreviations

ACE-27: Adult Comorbidity Evaluation-27
AHRR: Aryl hydrocarbon receptor repressor
AUC: Area under the curve
BMI: Body mass index
CpG: Cytosine-phosphate-guanine
CI: Confidence interval
DNAm: DNA methylation
EWAS: Epigenome-wide association study
GLM: Generalised linear model
HN5000: Head and Neck 5000
HNC: Head and neck cancer
HPV: Human papillomavirus
HR: Hazard ratio
ICD: International Classification of Diseases
IQR: Inter-quartile range
LASSO: Least absolute shrinkage and selection operator
MAR: Missing at random
MCAR: Missing completely at random
MI: Multiple imputation
NHS: National Health Service
OPC: Oropharyngeal cancer
QC: Quality control
ROC: Receiver-operator characteristic
RTOG: Radiation Therapy Oncology Group
SD: Standard deviation
