# Supplementary material for: Epigenetic prediction of complex traits and mortality in a cohort of individuals with oropharyngeal cancer
Source: Clin Epigenetics. 2020 Apr 22;12:58. doi: 10.1186/s13148-020-00850-4 (PMC7178612; doi:10.1186/s13148-020-00850-4)
Supplement: Supplementary file 1 — Additional file 1: Supplementary Table 1: Baseline descriptive characteristics of included participants, stratified by HPV status. Supplementary Table 2: Multivariable Cox proportional hazards results for model 2 (clinical) and model 3 (respective phenotype). Supplementary Table 3: Baseline descriptive characteristics of participants included in the sensitivity analysis (n=248). Supplementary Table 4: Results of the sensitivity analysis restricted to participants with data available for BMI. Supplementary Table 5: A comparison of minimally adjusted and fully adjusted Cox proportional hazards models results, using the imputed dataset (n=408). Supplementary Table 6: Details of array type and sample size for studies used to derive DNAm scores in this analysis. Supplementary Table 7: Proportion of missing data (n=408). [file 13148_2020_850_MOESM1_ESM.docx]

**Supplementary Table 1**: Baseline descriptive characteristics of included participants, stratified by HPV status

|  | **HPV-negative n=109** | | **HPV-positive n=255** | |  |  |
| --- | --- | --- | --- | --- | --- | --- |
| **Variable** | **N** | **Frequency** | **N** | **Frequency** | **Total** | ***p*-value*** |
| Gender |  |  |  |  |  | 0.589 |
| Male | 87 | 79.8% | 197 | 77.3% | 284 |  |
| Female | 22 | 20.2% | 58 | 22.7% | 80 |  |
| Age at enrolment |  |  |  |  |  | 0.216 |
| < 44 | 4 | 3.7% | 19 | 7.5% | 23 |  |
| 45 to 54 | 27 | 24.8% | 78 | 30.6% | 105 |  |
| 55 to 64 | 46 | 42.2% | 101 | 39.6% | 147 |  |
| 65 to 74 | 23 | 21.1% | 47 | 18.4% | 70 |  |
| 75 + | 9 | 8.3% | 10 | 3.9% | 19 |  |
| TNM stage |  |  |  |  |  | <0.001 |
| I | 9 | 8.3% | 7 | 2.7% | 16 |  |
| II | 17 | 15.6% | 14 | 5.5% | 31 |  |
| III | 21 | 19.3% | 31 | 12.2% | 52 |  |
| IV | 62 | 56.9% | 203 | 79.6% | 265 |  |
| BMI group |  |  |  |  |  | 0.003 |
| Not overweight (BMI ≤25) | 43 | 39.4% | 61 | 23.9% | 104 |  |
| Overweight or obese (BMI >25) | 66 | 60.6% | 194 | 76.1% | 260 |  |
| Comorbidity |  |  |  |  |  | <0.001 |
| None | 44 | 40.4% | 154 | 60.4% | 198 |  |
| Mild | 35 | 32.1% | 67 | 26.3% | 102 |  |
| Moderate/Severe | 30 | 27.5% | 34 | 13.3% | 64 |  |
| Education level |  |  |  |  |  | 0.918 |
| School education | 50 | 45.9% | 111 | 43.5% | 161 |  |
| College | 42 | 38.5% | 103 | 40.4% | 145 |  |
| Degree | 17 | 15.6% | 41 | 16.1% | 58 |  |
| Self-reported smoking status |  |  |  |  |  | <0.001 |
| Never | 17 | 15.6% | 90 | 35.3% | 107 |  |
| Former | 46 | 42.2% | 143 | 56.1% | 189 |  |
| Current | 46 | 42.2% | 22 | 8.6% | 68 |  |
| Self-reported alcohol intake |  |  |  |  |  | 0.038 |
| Non-drinker | 29 | 26.6% | 68 | 26.7% | 97 |  |
| Moderate | 16 | 14.7% | 67 | 26.3% | 83 |  |
| Hazardous-harmful | 64 | 58.7% | 120 | 47.1% | 184 |  |

**Abbreviations:** N, number; HR, hazard ratio; ll, lower confidence interval; ul, upper confidence interval. * *p*-value for difference.

**Supplementary Table 2:** Multivariable Cox proportional hazards results for model 2 (clinical) and model 3 (respective phenotype)

|  |  | Model 2* | | | |  | Model 3** | | | |
| --- | --- | --- | --- | --- | --- | --- | --- | --- | --- | --- |
| **Exposure** | **N** | **HR** | **ll** | **ul** | **p-value** |  |  |  |  |  |
| Self-reported phenotype | | | | | |  |  |  |  |  |
| Ever- vs. never-smoker | 364 | 2.47 | 1.29 | 4.72 | **0.006** |  |  |  |  |  |
| Hazardous to harmful drinker vs. not | 364 | 1.47 | 0.95 | 2.27 | 0.084 |  |  |  |  |  |
| Higher education vs. school education | 364 | 0.81 | 0.53 | 1.22 | 0.306 |  |  |  |  |  |
| BMI | 248 | 0.96 | 0.89 | 1.02 | 0.169 |  |  |  |  |  |
| DNAm score | | | | | | | | | | |
| *Smoking* |  |  |  |  |  |  |  |  |  |  |
| McCartney smoking (233 CpG sites) | 364 | 1.34 | 1.07 | 1.67 | **0.011** |  | 1.05 | 0.79 | 1.41 | 0.726 |
| *AHRR* (cg05575921) | 364 | 0.66 | 0.52 | 0.83 | **4.11E-04** |  | 0.79 | 0.58 | 1.07 | 0.125 |
| Joehanes (FDR) (18670 CpG sites) | 364 | 1.59 | 1.22 | 2.08 | **6.11E-04** |  | 1.35 | 0.99 | 1.84 | 0.056 |
| Joehanes (Bonferroni) (2623 CpG sites) | 364 | 1.59 | 1.26 | 2.00 | **9.82E-05** |  | 1.38 | 1.04 | 1.83 | **0.025** |
| Trejo Bayesian smoking (59 CpG sites) | 364 | 1.51 | 1.2 | 1.91 | **4.34E-04** |  | 1.26 | 0.93 | 1.72 | 0.14 |
| Zhang (4 CpG sites) | 364 | 1.38 | 1.1 | 1.74 | **6.26E-03** |  | 1.28 | 1.02 | 1.6 | **0.036** |
| *Alcohol consumption* |  |  |  |  |  |  |  |  |  |  |
| Liu Model 1 (5 CpG sites) | 364 | 1.25 | 1.03 | 1.51 | **0.023** |  | 1.19 | 0.97 | 1.47 | 0.094 |
| Liu Model 2 (23 CpG sites) | 364 | 1.17 | 0.96 | 1.42 | 0.126 |  | 1.1 | 0.89 | 1.36 | 0.357 |
| Liu Model 3 (78 CpG sites) | 364 | 1.25 | 1.05 | 1.49 | **0.014** |  | 1.2 | 0.99 | 1.45 | 0.067 |
| Liu Model 4 (144 CpG sites) | 364 | 1.26 | 1.06 | 1.49 | **9.99E-03** |  | 1.21 | 1.00 | 1.46 | 0.052 |
| McCartney alcohol (450 CpG sites) | 364 | 1.26 | 1.00 | 1.57 | **0.046** |  | 1.2 | 0.94 | 1.52 | 0.144 |
| *BMI* |  |  |  |  |  |  |  |  |  |  |
| McCartney BMI (1109 CpG sites) | 364 | 0.82 | 0.66 | 1.02 | 0.075 |  | 0.77 | 0.57 | 1.04 | 0.093 |
| Trejo Bayesian BMI (144 CpG sites) | 364 | 0.77 | 0.61 | 0.97 | **0.025** |  | 0.78 | 0.56 | 1.08 | 0.132 |
| *Educational attainment* |  |  |  |  |  |  |  |  |  |  |
| McCartney education (373 CpG sites) | 364 | 0.87 | 0.67 | 1.11 | 0.260 |  | 0.87 | 0.68 | 1.12 | 0.27 |

**Abbreviations:** N, number; HR, hazard ratio; ll, lower confidence interval; ul, upper confidence interval. * Adjusted for age, gender, TNM stage, HPV status and comorbidity (plus cell count and batch effects for epigenetic (DNAm models)). ** additionally, adjusted for the respective self-reported phenotype.

**Supplementary Table 3:** Baseline descriptive characteristics of participants included in the sensitivity analysis (n=248).

|  | **Alive (n=192)** | | **Dead (n=56)** | |  |
| --- | --- | --- | --- | --- | --- |
| Characteristic | N | Frequency | N | Frequency | p-value |
| **Gender** |  |  |  |  |  |
| Male | 147 | 76.6% | 45 | 80.4% |  |
| Female | 45 | 23.4% | 11 | 19.6% | 0.550 |
| **Age at enrolment** |  |  |  |  |  |
| < 44 | 16 | 8.3% | 1 | 1.8% |  |
| 45 to 54 | 61 | 31.8% | 16 | 28.6% |  |
| 55 to 64 | 76 | 39.6% | 17 | 30.4% |  |
| 65 to 74 | 32 | 16.7% | 14 | 25.0% |  |
| 75 + | 7 | 3.6% | 8 | 14.3% | 0.009 |
| **TNM stage** |  |  |  |  |  |
| Low | 29 | 15.1% | 7 | 12.5% |  |
| High | 163 | 84.9% | 49 | 87.5% | 0.626 |
| **HPV status** |  |  |  |  |  |
| Negative | 43 | 22.4% | 31 | 55.4% |  |
| Positive | 149 | 77.6% | 25 | 44.6% | <0.001 |
| **BMI group** |  |  |  |  |  |
| not overweight | 73 | 38.0% | 31 | 55.4% |  |
| overweight or obese | 119 | 62.0% | 25 | 44.6% | 0.021 |
| **Comorbidity** |  |  |  |  |  |
| None | 119 | 62.0% | 25 | 44.6% |  |
| Mild | 51 | 26.6% | 18 | 32.1% |  |
| Moderate/Severe | 22 | 11.5% | 13 | 23.2% | 0.031 |
| **Education level** |  |  |  |  |  |
| School education | 84 | 43.8% | 30 | 53.6% |  |
| College | 80 | 41.7% | 20 | 35.7% |  |
| Degree | 28 | 14.6% | 6 | 10.7% | 0.414 |
| **Self-reported smoking status** |  |  |  |  |  |
| Never | 72 | 37.5% | 7 | 12.5% |  |
| Former | 98 | 51.0% | 29 | 51.8% |  |
| Current | 22 | 11.5% | 20 | 35.7% | <0.001 |
| **Self-reported alcohol intake** |  |  |  |  |  |
| Non-drinker | 53 | 27.6% | 15 | 26.8% |  |
| Moderate | 47 | 24.5% | 9 | 16.1% |  |
| Hazardous-harmful | 92 | 47.9% | 32 | 57.1% | 0.349 |

**Abbreviations:** BMI, body mass index; HPV, human papillomavirus; N, number.

**Supplementary Table 4**: Results of the sensitivity analysis.

|  |  | Minimally adjusted | |  |  |  |  | Fully adjusted | |  |  |
| --- | --- | --- | --- | --- | --- | --- | --- | --- | --- | --- | --- |
| **Exposure** | **N** | **HR** | **ll** | **ul** | **p-value** |  | **N** | **HR** | **ll** | **ul** | **p-value** |
| Self-reported phenotype | | | | | | | | | | | |
| Ever vs. never smoker | 248 | 3.64 | 1.65 | 8.07 | **1.42E-03** |  | 248 | 2.47 | 1.07 | 5.73 | **0.035** |
| Hazardous to harmful drinker vs. not | 248 | 1.48 | 0.86 | 2.56 | 0.161 |  | 248 | 1.24 | 0.70 | 2.21 | 0.458 |
| Higher education vs school education | 248 | 0.74 | 0.44 | 1.25 | **0.026** |  | 248 | 0.94 | 0.54 | 1.64 | 0.837 |
| BMI | 248 | 0.93 | 0.87 | 0.99 | **0.028** |  | 248 | 0.98 | 0.92 | 1.06 | 0.655 |
| DNAm score | | | | | | | | | | | |
| McCartney smoking (233 CpG sites) | 248 | 1.49 | 1.13 | 1.97 | **4.31E-03** |  | 248 | 1.32 | 0.97 | 1.81 | 0.076 |
| McCartney alcohol (450 CpG sites) | 248 | 1.31 | 0.98 | 1.76 | 0.067 |  | 248 | 1.04 | 0.71 | 1.51 | 0.85 |
| McCartney BMI (1109 CpG sites) | 248 | 0.76 | 0.57 | 1.01 | 0.059 |  | 248 | 0.77 | 0.57 | 1.04 | 0.093 |
| McCartney education (373 CpG sites) | 248 | 0.86 | 0.65 | 1.14 | 0.294 |  | 248 | 0.94 | 0.69 | 1.28 | 0.692 |
| Liu Model 1 alcohol (5 CpG sites) | 248 | 1.36 | 1.08 | 1.73 | **9.39E-03** |  | 248 | 1.43 | 1.07 | 1.92 | **0.017** |
| Liu Model 2 alcohol (23 CpG sites) | 248 | 1.33 | 1.03 | 1.72 | 0.294 |  | 248 | 1.33 | 0.98 | 1.80 | 0.068 |
| Liu Model 3 alcohol (78 CpG sites) | 248 | 1.23 | 1.02 | 1.49 | **0.028** |  | 248 | 1.32 | 1.03 | 1.69 | **0.027** |
| Liu Model 4 alcohol (144 CpG sites) | 248 | 1.22 | 1.01 | 1.46 | **0.037** |  | 248 | 1.29 | 1.02 | 1.63 | **0.036** |
| *AHRR* smoking (cg05575921) | 248 | 0.63 | 0.47 | 0.83 | **1.28E-03** |  | 248 | 0.89 | 0.60 | 1.32 | 0.551 |
| Joehanes (FDR) smoking (18670 CpG sites) | 248 | 1.84 | 1.36 | 2.49 | **7.43E-05** |  | 248 | 1.59 | 1.09 | 2.32 | **0.016** |
| Joehanes (Bonferroni) smoking (2623 CpG sites) | 248 | 1.72 | 1.32 | 2.24 | **5.24E-05** |  | 248 | 1.50 | 1.06 | 2.12 | **0.022** |
| Zhang smoking (4 CpG sites) | 248 | 1.41 | 1.04 | 1.91 | **0.029** |  | 248 | 1.33 | 1.00 | 1.77 | **0.047** |
| Trejo Bayesian smoking (59 CpG sites) | 248 | 1.61 | 1.21 | 2.14 | **1.17E-03** |  | 248 | 1.12 | 0.75 | 1.67 | 0.582 |
| Trejo Bayesian BMI (144 CpG sites) | 248 | 0.76 | 0.59 | 0.99 | **0.045** |  | 248 | 0.77 | 0.56 | 1.08 | 0.132 |

**Abbreviations:** N, number; HR, hazard ratio; ll, lower confidence interval; ul, upper confidence interval. *Self-reported phenotypes adjusted for age and gender; risk scores adjusted for age, gender, cell counts and batch effects. **phenotypes additionally adjusted for clinical variables (TNM stage, HPV status, co-morbidity and BMI) and a combination of smoking, alcohol intake, education and BMI, as appropriate to the model; risk scores additionally adjusted for clinical variables and corresponding phenotype.

**Supplementary Table 5:** A comparison of minimally adjusted and fully adjusted Cox proportional hazards models results, using the imputed dataset (n=408).

|  | Minimally adjusted* |  |  |  |  | Fully adjusted** |  |  |  |
| --- | --- | --- | --- | --- | --- | --- | --- | --- | --- |
| **Exposure** | **HR** | **ll** | **ul** | **p-value** |  | **HR** | **ll** | **ul** | **p-value** |
| Self-reported phenotypes | | | | | | | | | |
| Ever- vs. never-smoker | 3.65 | 1.95 | 6.82 | **5.20E-05** |  | 2.56 | 1.33 | 4.92 | **4.94E-03** |
| Hazardous to harmful drinker vs. not | 1.49 | 1.00 | 2.23 | 0.052 |  | 1.23 | 0.81 | 1.86 | 0.328 |
| Higher education vs. school education | 0.83 | 0.57 | 1.22 | 0.338 |  | 1.01 | 0.97 | 1.06 | 0.594 |
| BMI | 0.97 | 0.93 | 1.01 | 0.117 |  | 0.93 | 0.63 | 1.36 | 0.693 |
| Epigenetic scores | | | | | | | | | |
| McCartney smoking (233 CpG sites) | 1.25 | 1.02 | 1.53 | **0.030** |  | 1.08 | 0.83 | 1.41 | 0.554 |
| McCartney alcohol (450 CpG sites) | 1.51 | 1.23 | 1.85 | **6.15E-05** |  | 1.14 | 0.92 | 1.41 | 0.244 |
| McCartney BMI (1109 CpG sites) | 0.83 | 0.68 | 1.01 | 0.061 |  | 0.84 | 0.67 | 1.04 | 0.114 |
| McCartney education (373 CpG sites) | 0.80 | 0.65 | 0.99 | **0.044** |  | 0.91 | 0.72 | 1.14 | 0.398 |
| Liu Model 1 alcohol (5 CpG sites) | 1.31 | 1.10 | 1.55 | **1.99E-03** |  | 1.16 | 0.95 | 1.41 | 0.140 |
| Liu Model 2 alcohol (23 CpG sites) | 1.25 | 1.04 | 1.50 | **0.018** |  | 1.07 | 0.87 | 1.31 | 0.526 |
| Liu Model 3 alcohol (78 CpG sites) | 1.23 | 1.06 | 1.42 | **7.56E-03** |  | 1.14 | 0.95 | 1.36 | 0.153 |
| Liu Model 4 alcohol (144 CpG sites) | 1.20 | 1.04 | 1.39 | **0.012** |  | 1.13 | 0.95 | 1.35 | 0.169 |
| *AHRR* smoking (cg05575921) | 0.59 | 0.48 | 0.72 | **2.92E-07** |  | 0.74 | 0.56 | 0.98 | **0.033** |
| Joehanes (FDR) smoking (18670 CpG sites) | 1.65 | 1.33 | 2.06 | **6.65E-06** |  | 1.34 | 1.02 | 1.76 | **0.037** |
| Joehanes (Bonferroni) smoking (2623 CpG sites) | 1.66 | 1.37 | 2.01 | **2.95E-07** |  | 1.39 | 1.08 | 1.80 | **0.012** |
| Zhang smoking (4 CpG sites) | 1.49 | 1.19 | 1.86 | **4.57E-04** |  | 1.28 | 1.04 | 1.58 | **0.022** |
| Trejo Bayesian smoking (59 CpG sites) | 1.70 | 1.38 | 2.08 | **4.24E-07** |  | 1.29 | 0.98 | 1.71 | 0.073 |
| Trejo Bayesian BMI (144 CpG sites) | 0.74 | 0.61 | 0.91 | **4.63E-03** |  | 0.72 | 0.56 | 0.91 | **7.24E-03** |

**Abbreviations:** N, number; HR, hazard ratio; ll, lower confidence interval; ul, upper confidence interval. *Self-reported phenotypes adjusted for age and gender; epigenetic scores adjusted for age, gender, cell counts and batch effects. **phenotypes additionally adjusted for clinical variables (TNM stage, HPV status and co-morbidity), and a combination of smoking, alcohol intake, education and BMI, as appropriate to the model; risk scores additionally adjusted for clinical variables, the corresponding phenotype predicted by the score of interest and the remaining self-reported phenotypes (excluding BMI).

**Supplementary Table 6**: Details of array type and sample size for studies used to derive DNAm scores in this analysis

| Contributing study | Array used | Phenotype | Sample size (discovery) |
| --- | --- | --- | --- |
| Joehanes et al. | Illumina 450K | Smoking | 9,389 (2,433 current smokers, 6,956 never smokers) |
| Liu et al. | Illumina 450K | Alcohol consumption | 9,643 |
| McCartney et al. | Illumina MethylationEPIC | Alcohol consumption | 2,819 |
|  |  | BMI | 5,087 |
|  |  | Educational attainment | 4,804 |
|  |  | Smoking | 3,444 (921 current smokers, 2,523 never smokers) |
| Trejo et al. | Illumina MethylationEPIC | BMI | 5,087 |
|  |  | Smoking | 3,444 (921 current smokers, 2,523 never smokers) |
| Zhang et al. | Illumina 450K | Smoking | 500 (89 current smokers, 163 former smokers, 233 never smokers) |

**Supplementary Table 7:** Proportion of missing data (n=408).

| **Variable** | **Missing** | **% Missing** |
| --- | --- | --- |
| Age | 5 | 1.2 |
| Gender | 0 | 0 |
| TNM | 0 | 0 |
| HPV status | 0 | 0 |
| Comorbidity | 3 | 0.7 |
| BMI | 136 | 33.3 |
| Education | 19 | 4.7 |
| Smoking status | 16 | 3.9 |
| Alcohol consumption | 8 | 2.0 |
