# Supplementary material for: Epigenetic prediction of complex traits and mortality in a cohort of individuals with oropharyngeal cancer
Source: Clin Epigenetics. 2020 Apr 22;12:58. doi: 10.1186/s13148-020-00850-4 (PMC7178612; doi:10.1186/s13148-020-00850-4)
Supplement: Supplementary file 2 — Additional file 2: Supplementary Figure 1a: Kaplan-Meier survival curves based on demographic and clinical covariates. Comorbidity categories were defined according to the severity or organ decompensation: none (coded 0), mild (coded 1), moderate (coded 2), or severe (coded 3). See text for more details. Supplementary Figure 1b: Kaplan-Meier survival curves based on our phenotypes of interest. Supplementary Figure 2: The association between variance explained by DNAm score and hazard ratio for 4-year mortality (Model 3). Hazard ratios are plotted as absolute log-transformed values for comparability. Supplementary Figure 3: ROC curves detailing the predictive accuracy of DNAm risk scores, self-reported phenotype and a combination of the two, against ~4-year mortality (median 3.9 years) in HN5000. ROC curves are provided for smoking, alcohol consumption, BMI and educational attainment. DNAm AUCs reflect use of the DNAm scores for these phenotypes which explained the greatest phenotypic variance: smoking = Trejo Bayesian model, alcohol consumption = Liu et al. model 4, BMI = Trejo Bayesian model, educational attainment = McCartney LASSO model. Abbreviations: AUC, area under curve; DNAm, DNA methylation; ROC, receiver-operator curve [file 13148_2020_850_MOESM2_ESM.docx]

**Supplementary Figure 1a:** Kaplan-Meier survival curves based on demographic and clinical covariates. Comorbidity categories were defined according to the severity or organ decompensation: none (coded 0), mild (coded 1), moderate (coded 2), or severe (coded 3). See text for more details.

**Supplementary Figure 1b**: Kaplan-Meier survival curves based on our phenotypes of interest.

**Supplementary Figure 2**: The association between variance explained by DNAm and hazard ratio for 4-year mortality (Model 3). Hazard ratios are plotted as absolute log-transformed values for comparability.

**Supplementary Figure 3:** ROC curves detailing the predictive accuracy of DNAm risk scores, self-reported phenotype and a combination of the two, against ~4-year mortality (median 3.9 years) in HN5000. ROC curves are provided for smoking, alcohol consumption, BMI and educational attainment. DNAm AUCs reflect use of the DNAm scores for these phenotypes which explained the greatest phenotypic variance: smoking = Trejo Bayesian model, alcohol consumption = Liu et al. model 4, BMI = Trejo Bayesian model, educational attainment = McCartney LASSO model. See text for more details.


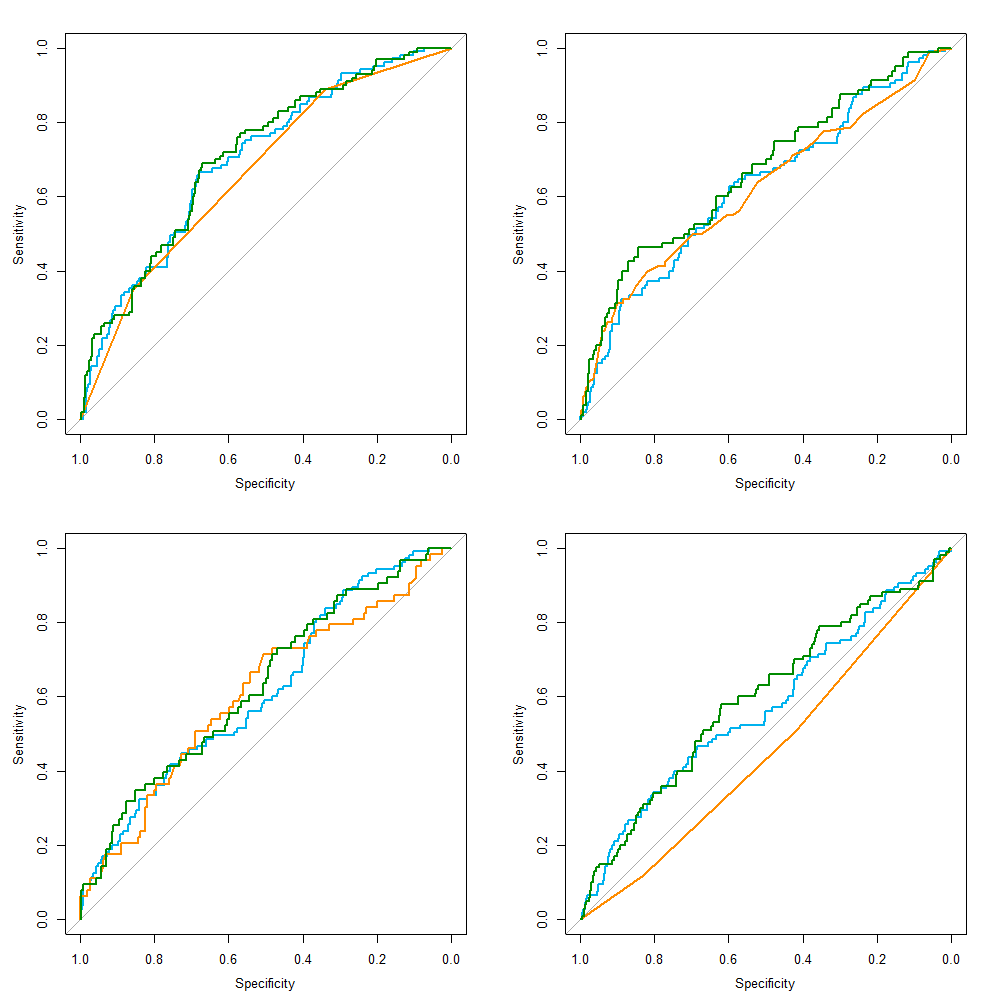


Smoking

Alcohol consumption

DNAm AUC: 0.698

DNAm AUC: 0.627

Phenotype AUC: 0.666

Phenotype AUC: 0.622

DNAm + Phenotype AUC: 0.708

DNAm + Phenotype AUC: 0.669

**
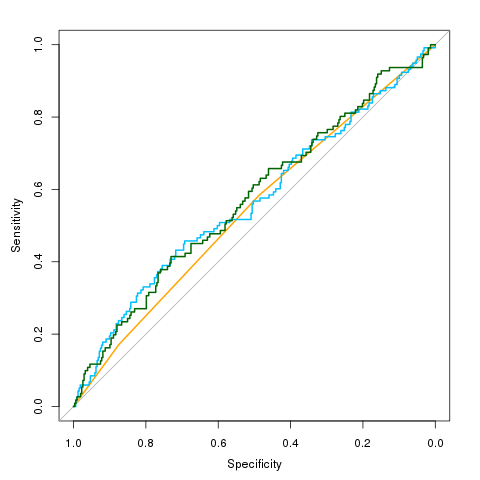
**

BMI

Educational attainment

DNAm AUC: 0.613

DNAm AUC: 0.565

Phenotype AUC: 0.608

Phenotype AUC: 0.543

DNAm + Phenotype AUC: 0.629

DNAm + Phenotype AUC: 0.572

**Abbreviations:** AUC, area under curve; DNAm, DNA methylation; ROC, receiver-operator curve

**Supplementary Figure 4:** Directed acyclic graph (DAG) illustrating a mediator (M) on the pathway between phenotype (X) and mortality (Y).

X

Y

M

C

U

*Unlike adjustment for a potential confounder C, adjusting for M would represent an over-adjustment as it is on the causal pathway between X and Y, and could induce a spurious association in the presence of a mediator-outcome confounder (U).*

Legend

Alcohol DNAm predictor

BMI DNAm predictor

Educational attainment DNAm predictor

Smoking DNAm predictor
